# Insights Into Cockayne Syndrome Type B: What Underlies Its Pathogenesis?

**DOI:** 10.1111/acel.70136

**Published:** 2025-06-19

**Authors:** Ricardo Afonso‐Reis, Cristiana R. Madeira, David V. C. Brito, Clévio Nóbrega

**Affiliations:** ^1^ ABC‐RI Algarve Biomedical Center Research Institute Faro Portugal; ^2^ Faculdade de Medicina e Ciências Biomédicas Universidade do Algarve Faro Portugal; ^3^ Horae Gene Therapy Center UMass Chan Medical School Worcester Massachusetts USA; ^4^ Programa doutoral em Ciências Biomédicas Universidade do Algarve Faro Portugal; ^5^ ABC Collaborative Laboratory: Integrated Ageing and Rejuvenation Solutions (ABC CoLAB–Ageing Better) Loulé Portugal

**Keywords:** accelerated aging, cockayne syndrome type B, DNA damage repair, mitochondrial dysfunction, progeroid syndrome, transcription impairment

## Abstract

Cockayne Syndrome (CS) is an autosomal recessive disorder arising from mutations in either of two disease‐associated genes, *ERCC6 or ERCC8*. CS patients exhibit cutaneous photosensitivity, neuropathological abnormalities, severe growth retardation, a distinctive facial appearance with pronounced sunken eyes, musculoskeletal anomalies, sensory impairment, and dental decay. Approximately 70% of all CS cases carry *ERCC6* mutations; therefore, this review will focus solely on Cockayne Syndrome complementation group B (CS‐B). CS‐B exhibits several hallmarks of aging, including genomic instability, epigenetic modifications, loss of proteostasis, and mitochondrial failure. CS‐B is proposed to result from the accumulation of DNA damage and the resulting transcription impairment. However, the main pathophysiological mechanisms underlying the severe cellular impairments observed in CS‐B remain unclear. Here, we review the current literature to elucidate ERCC6‐related mechanisms, highlighting the key and emerging pathological mechanisms underlying CS‐B, as well as their putative interactions. Considering the mechanisms that heavily rely on ERCC6, we propose that CS‐B pathogenesis arises from a combination of DNA damage accumulation, transcriptional dysregulation, and mitochondrial dysfunction. Furthermore, we argue that these molecular features influence each other, rather than acting as isolated mechanisms. This suggests that the crosstalk between mechanisms is a key factor for CS‐B pathogenesis. Although efforts have been made to unveil CS‐B pathogenesis, research is still lacking, hindering progress in understanding this deadly disease. Future work will prove crucial to determine the main contributor to CS‐B pathogenesis and identify new interactions between CS‐B‐affected mechanisms.

## Graphical abstract

Cockayne Syndrome complementation group B (CS‐B) is a highly debilitating progeroid syndrome that often culminates in the death of patients before adulthood. This review explores the pathogenesis of CS‐B and proposes that a combination of DNA damage accumulation, transcriptional dysregulation, and mitochondrial dysfunction is its underlying cause.

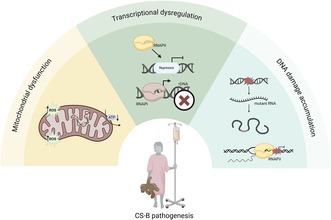

## 1 Introduction

The first clinical report of a Cockayne syndrome (CS) patient dates back to 1936 (Cockayne 1936). This diagnosis was based on phenotypical characteristics, as technology was limited to perform genetic testing. A clear molecular diagnosis of patients was only achieved once the two disease‐causing genes were identified, allowing later genetic confirmation (Henning et al. 1995; Troelstra et al. 1992). This discovery was prompted by uncovering CS patient cells failure to restore RNA synthesis following ultraviolet (UV) radiation exposure (Mayne and Lehmann 1982; Schmickel et al. 1977).

Cockayne Syndrome is an autosomal recessive disorder that arises from mutations in one of two distinct genes: (i) *excision repair cross‐complementing protein group 6* (*ERCC6*/*CSB*) or *excision repair cross‐complementing protein group 8* (*ERCC8*/*CSA*), located in chromosomes 10q11 and 5q11, respectively (Tiwari et al. 2021). An alternative form of CS, termed Xeroderma Pigmentosum (XP)‐CS, has also been described. It arises from mutations in either *ERCC3*, *ERCC4*, *ERCC1*, *ERCC5*, or *ERCC2* leading to both CS and XP features (Rapin et al. 2000; Vessoni et al. 2020).

This review will focus solely on Cockayne Syndrome complementation group B (CS‐B), considering that approximately 70% of CS patients have a mutation in the *ERCC6* gene (Laugel 2013). Furthermore, *ERCC6* mutations manifest into more severe forms of CS with prenatal onset. Conversely, *ERCC8* mutations are associated with mild or moderate phenotypes (Jaarsma et al. 2013; Laugel 2013).

### 1.1 CS Clinical Features

The key clinical manifestations of CS encompass cutaneous photosensitivity, intellectual disability, severe growth retardation, distinctive facial appearance with pronounced sunken eyes, sensory impairment, skeletal anomalies, and dental decay (Cockayne 1936; Laugel 2013; Nance and Berry 1992; Vélez‐Cruz and Egly 2013; Wilson et al. 2016). Most subtypes of CS exhibit these clinical manifestations, with varying degrees of severity, that consistently worsen overtime. The clinical heterogeneity of CS reflects multiple impaired systems in this disorder and is a critical element contributing to its devastating progression.

CS patients have varied disease onset and progression rates/severity; however, the clinical manifestations are consistent among patients. A clinical diagnostic criteria based on age of onset and severity was established to address this issue, categorizing CS into three subtypes (Nance and Berry 1992). These subtypes do not denote distinct clinical subgroups; rather, they indicate positions within a spectrum of clinical manifestation, characterized by overlapping groups and no definitive separation threshold (Laugel 2013). Type I, or moderate CS, exhibits classical disease progression with symptoms manifesting by the age of 1–2, and patients have a mean life expectancy of roughly 16 years. Type II is the congenital variant of CS with a mean life expectancy of 5–6 years. Finally, the onset of disease in type III, or mild CS, occurs several years after birth, with an average life expectancy of 30 years. Notably, CS severity is directly correlated with the age of disease onset (Laugel 2013; Nance and Berry 1992; Natale 2011).

Two non‐canonical CS subtypes have been proposed at either end of the clinical spectrum, ranging from the most severe to the mildest: cerebro‐oculo‐facio‐skeletal syndrome (COFS) and UV‐sensitive syndrome (UVSS) (Fujiwara et al. 1981; Laugel 2013; Pena and Shokeir 1974). These two types of CS‐related syndromes are rarely observed in CS patients. Nevertheless, while performing diagnostics within the clinical continuum, they should still be considered. COFS, the most severe subtype of CS, primarily arises from *ERCC6* mutations; however, cases of COFS associated with *ERCC2*, ERCC5, and *ERCC1* mutations have also been reported (Laugel et al. 2008). The initial symptoms manifest during gestation, leading to the most severe manifestations and an estimated life expectancy of about 4–5 years (Laugel et al. 2008; Pena and Shokeir 1974). Conversely, UVSS, initially characterized independently of CS, is proposed to denote the mildest subtype of CS. This subtype is associated with mutations in *ERCC6*, *ERCC8* and UVstimulated scaffold protein A (*UVSSA*) primarily characterized by cutaneous photosensitivity, with no further clinical manifestations or only adult onset of additional symptoms (Horibata et al. 2004; Nardo et al. 2009; Zhang et al. 2012). COFS and UVSS have been regarded as additional variants of CS, nevertheless, this is still disputed, as they do not conform to the current established diagnostic criteria for CS (Laugel 2013). Regardless of disease severity, death is primarily attributed to pneumonia/respiratory complications, and less frequently to kidney failure (Natale 2011).

CS affects both male and female from all ethnicities in an equitable manner and displays a prevalence of approximately 2.7 births per million throughout Western Europe and Japan (Kleijer et al. 2008; Kubota et al. 2015).

### 1.2 Genetics of CS‐B

**FIGURE 1:**
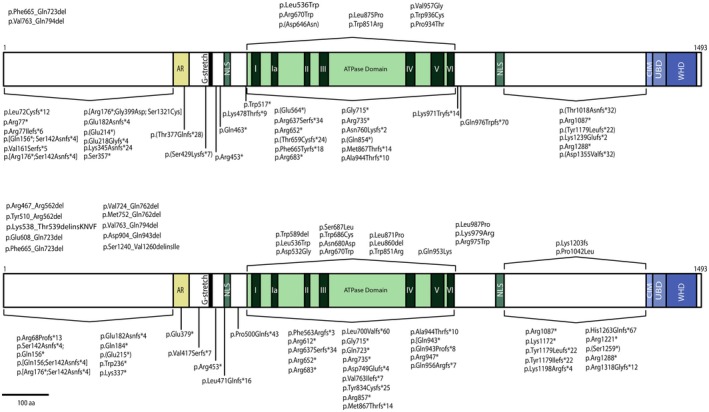
ERCC6 protein structure and mutations location. ERCC6 protein and regions of interest are made to scale. Acidic region (AR); Glutamine stretch (G‐stretch); nuclear localization signal (NLS); ERCC8 interaction motif (CIM); ubiquitin‐binding domain (UBD); winged‐helix domain (WHD). *ERCC6* homozygous (A) and heterozygous (B) mutations are represented in their relative position in the ERCC6 protein. Missense mutations are shown above the protein, while nonsense mutations are represented below the protein. Insertions/deletions are not indicated in their relative position and are found in the top left corner (A, B). Stop codon is represented by (*). Mutation nomenclature represented according to HGVS recommendations.

Currently, there have been reported a total of 105 homozygous and heterozygous mutations associated with *ERCC6* (listed in Table S1 and protein location in the ERCC6 represented in Figure 1) (Duong et al. 2022; Lin et al. 2021; Vessoni et al. 2020; Zhang et al. 2020). Premature STOP codon mutations account for the majority of *ERCC6* mutations, totaling 68 occurrences, followed by 16 missense mutations, 11 deletions, and 10 composite mutations (Table S1). Concerning mutation location, *ERCC6* mutations predominantly occur in the region encoding the ATPase domain, with more than 50% of CS‐B cases exhibiting at least one allele with mutation in this region (Table S1).

There is no consensus regarding the relationship between genotype and disease phenotype of CS‐B, as well as onset, that has yet to be established, due to conflicting clinical observations. Several genotype–phenotype correlations have been proposed, including a relationship between the number of downstream mutations and a more severe CS‐B phenotype, as well as a link of specific mutations with a more severe phenotype (Damaj‐Fourcade et al. 2022; He, Sun, et al. 2017; Kou et al. 2018; Laugel 2013). Conversely, cases have been reported where identical mutations result in remarkably different clinical manifestations, highlighting the complexity and variability of the disease. For example, three patients harboring identical *ERCC6* mutations displayed high levels of clinical heterogeneity with distinct clinical features (Zayoud et al. 2021). The clinical differences noted among CS‐B patients harboring identical mutations may be attributed to epigenetic regulation of *ERCC6* gene expression. *ERCC6* transcriptional repression, regardless of the patient's mutation, may lead to reduced ERCC6 availability, compromising critical molecular pathways. Particularly, hypoacetylation of histone H3 at the ERCC6 promoter causes ERCC6 depletion, subsequently causing mitochondrial dysfunction and replicative senescence (Crochemore et al. 2019). Additionally, UV‐B exposure triggers a coordinated epigenetic response resulting in hypermethylation and histone deacetylation of the ERCC6 promoter region (Wang et al. 2016). The relationship between ERCC6 epigenetic regulation and CS‐B phenotype is yet to be determined in detail. These epigenetic modulations provide a plausible explanation for the phenotypical differences, even between patients sharing identical mutations. Alongside genetic and epigenetic factors, lifestyle decisions may also significantly influence the severity and progression of the CS‐B phenotype, although this aspect would be more challenging to assess due to its complexity.

### 1.3 ERCC6 Protein

The ERCC6 protein consists of 1493 amino acids (aa), with a molecular weight of 168‐KDa and belongs to the SWI2/SNF2 family of chromatin remodeling helicases/ATPases (Troelstra et al. 1992; Vélez‐Cruz and Egly 2013). ERCC6 is structurally composed of three different segments: (i) the N‐terminal, (ii) the central segment, comprised mainly by the ATPase domain and (iii) the C‐terminal (Figure 1).

The N‐terminal features an acidic‐rich region (aa 356–394) with overall negative charge (Troelstra et al. 1992). This region is typically associated with protein–protein interactions, particularly between nuclear and DNA‐binding proteins, such as chromatin remodelers and transcription regulators (Carpenter et al. 2005; Vessoni et al. 2020). It has also been shown that the N‐terminal region is responsible for negatively regulating the association of ERCC6 with chromatin (Lake et al. 2010). Interestingly, several studies have shown that the acidic region of ERCC6 is not essential for DNA repair following UV exposure (Brosh Jr. et al. 1999; Lake et al. 2010; Sunesen et al. 2000).

The ATPase domain (aa 510–960) comprises seven helicase motifs conserved in DNA and RNA helicases, which are essential for DNA repair (Brosh Jr. et al. 1999). This domain confers ERCC6 with its key chromatin remodeling activity, as evidenced by the lack of chromatin remodeling activity in ATPase‐deficient ERCC6 (Citterio et al. 2000). ERCC6, as a member of the SWI2/SNF2 family, lacks helicase activity; instead, it alters nucleosome positioning and the interactions between DNA and histones through ATP hydrolysis. The restructuring process rearranges the DNA from a condensed state to an accessible state, facilitating access for transcription and DNA repair machinery to the underlying DNA (Citterio et al. 2000; Pazin and Kadonaga 1997; Selby and Sancar 1997). Additionally, ERCC6 exhibits strand‐annealing and exchange activity towards single‐stranded DNA, which are suggested to contribute to DNA repair and transcription; however, the significance of these activities in these processes remains unclear (Muftuoglu et al. 2006). Interestingly, ERCC6 ATPase activity exhibits variable significance across the different DNA repair mechanisms. The repair of DNA lesions induced by ultraviolet and ionizing radiation appears to rely heavily on ERCC6‐mediated ATP hydrolysis, whereas certain oxidative base lesions are independent of its ATPase activity (Batenburg et al. 2015; Selzer et al. 2002).

The C‐terminal contains a ubiquitin binding domain (UBD; aa 1400–1428) integrated within the winged‐helix domain (WHD; aa 1417–1493), essential for driving incision of UV‐induced DNA damage and the recruitment of ERCC6 to DNA double strand breaks (Anindya et al. 2010; Takahashi et al. 2019). The c‐terminus also possesses an ERCC8 interaction motif (CIM, aa 1385–1399), located upstream of the UBD, which is responsible for the recruitment of ERCC8 by ERCC6 to the DNA damage site during TC‐NER (van der Weegen et al. 2020).

ERCC6 has two nuclear localization signals (NLS) flanking the ATP domain, NLS1 (aa 466–481) and NLS2 (aa 1038–1055) (Iyama et al. 2018; Lange et al. 2007). A third NLS upstream of NLS1, named NLS3, and three putative nucleolar localization signals (NoLS), designated NoLS 1 to 3, are predicted through computational analysis (Iyama et al. 2018). NLS1 and NLS2 are responsible for ERCC6 nuclear localization, while nucleolar targeting is mostly attributed to the cooperation between NoLS1 and NLS1 (Iyama et al. 2018).

ERCC6 and ERCC8 exhibit functional cooperativity across diverse molecular pathways, suggesting that CS‐B and CS‐A share overlapping mechanistic deficiencies. However, mutations in *ERCC8* generally result in milder phenotypes compared to those arising from *ERCC6* mutations. This disparity suggests that ERCC6 may participate in additional molecular pathways independently of ERCC8, or alternatively, that ERCC6 fulfills a more pivotal function within their common processes. In line with this, briefly describing ERCC8 will provide a better understanding of ERCC6's role in both physiological and pathological contexts. The ERCC8 protein comprised of 396 amino acids with 44‐KDa, belongs to the “Trp‐Asp (WD) 40 repeat” family of structural and regulatory proteins (Henning et al. 1995). Despite lacking any described enzymatic function, ERCC8 is a component of the multi‐subunit E3 ubiquitin ligase complex, known as cullin 4‐RING ubiquitin ligase (CRL4). Consequently, through the integration into this complex, ERCC8 indirectly promotes the ubiquitination of ERCC6 and stalled RNAPII (Groisman et al. 2006; Henning et al. 1995; Nakazawa et al. 2020).

ERCC8 promotes ubiquitination of Nucleolin (Ncl), a rDNA synthesis regulator, and enhances ERCC6 binding to Ncl, which stimulates Ncl binding to rDNA (Okur, Lee, et al. 2020). Furthermore, ERCC8 cooperates with ERCC6 to promote RNA polymerase I transcription and subsequent ribosomal biogenesis (Koch et al. 2014; Okur, Lee, et al. 2020). Likewise, transcription dependent on activating transcription factor 3 (ATF3) is regulated by ubiquitin‐mediated proteasomal degradation of ATF3, promoted by ERCC8 and ERCC6 (Epanchintsev et al. 2017). The transcription regulation of neuronal genes has also been proposed to be regulated by ERCC6 and ERCC8 mediated ubiquitination and degradation of Necdin (Liang et al. 2023). ERCC8, in conjunction with ERCC6, indirectly regulates transcriptional programs involved in cell survival through the ubiquitination of p53, which leads to its subsequent degradation (Latini et al. 2011). Finally, the cooperation between ERCC6 and ERCC8 promotes the ubiquitination and subsequent degradation of PRC1, a key regulator of cytokinesis (Paccosi et al. 2020). In an ERCC6 independent manner, ERCC8 is involved in the ubiquitination of Cyclin B at the centrosome during mitosis (Paccosi et al. 2023). Similarly, only ERCC8 is suggested to be associated with the nuclear envelope integrity by modulating the formation of LEMD2‐lamin A/C complexes in the nuclear envelope (Yang et al. 2024).

In mitochondria, ERCC8 cooperates with ERCC6 to modulate the AMPK‐ULK1/DRP1 pathway, which is essential for mitochondrial homeostasis (Okur, Fang, et al. 2020). Furthermore, ERCC8 was found to interact, alongside ERCC6, with mitochondrial proteins involved in oxidative damage repair (Aamann et al. 2010; Kamenisch et al. 2010).

ERCC8 is also recruited to complex DNA lesions in the presence of ERCC6, including DNA interstrand crosslinks (ICLs) and double strand breaks DSBs. Nonetheless, the role of ERCC8 in the DNA repair mechanisms that resolve these lesions has not been elucidated (Iyama and Wilson 2016).

## 2 ERCC6 Functions

CS proteins have traditionally been associated with DNA repair mechanisms. This is attributed to the initial discovery of ERCC6 as a complementing factor of the DNA repair pathway, nucleotide excision repair (NER) (Troelstra et al. 1992). Currently, it is well established that ERCC6 is crucial in TC‐NER, a sub‐pathway of the DNA repair mechanism NER. TC‐NER is employed during transcription when a helix‐distorting lesion, typically caused by UV radiation, stalls RNAPII and subsequently arrests transcription until the lesion is repaired (Menck and Munford 2014). However, many clinical phenotypes of CS‐B cannot be solely attributed to impairment of TC‐NER, suggesting that ERCC6 is involved in other cellular pathways besides TC‐NER (Cleaver et al. 2013; Costanzo et al. 2024; Tiwari et al. 2021; Vessoni et al. 2020). Indeed, ERCC6 has been shown to participate in pathways beyond DNA repair, specifically transcription (Bradsher et al. 2002), ribosomal metabolism (Alupei et al. 2018), mitochondrial homeostasis (Okur, Fang, et al. 2020), and oxidative stress (D'Errico et al. 2013).

### 2.1 ERCC6 Role in DNA Integrity Maintenance

Cells receive tens of thousands of DNA lesions daily, due to the DNA intrinsic reactive properties, rendering it highly susceptible to damage (Chatterjee and Walker 2017). Such lesions may be sustained through a wide range of exogenous events, including exposure to UV or ionizing radiation (IR). Likewise, endogenous events such as errors linked to DNA polymerase activity during DNA replication or metabolites, namely reactive oxygen species (ROS), may result in DNA lesions (Jackson and Bartek 2009; Tiwari et al. 2021). To counter this, cells employ many mechanisms to detect and/or repair DNA damage, including those involving ERCC6, such as TC‐NER, BER (Tuo et al. 2002), DSBs repair (Batenburg et al. 2015) and ICLs repair (Enoiu et al. 2012).

#### 2.1.1 Transcription‐Coupled Nucleotide Excision Repair (TC‐NER)

**FIGURE 2:**
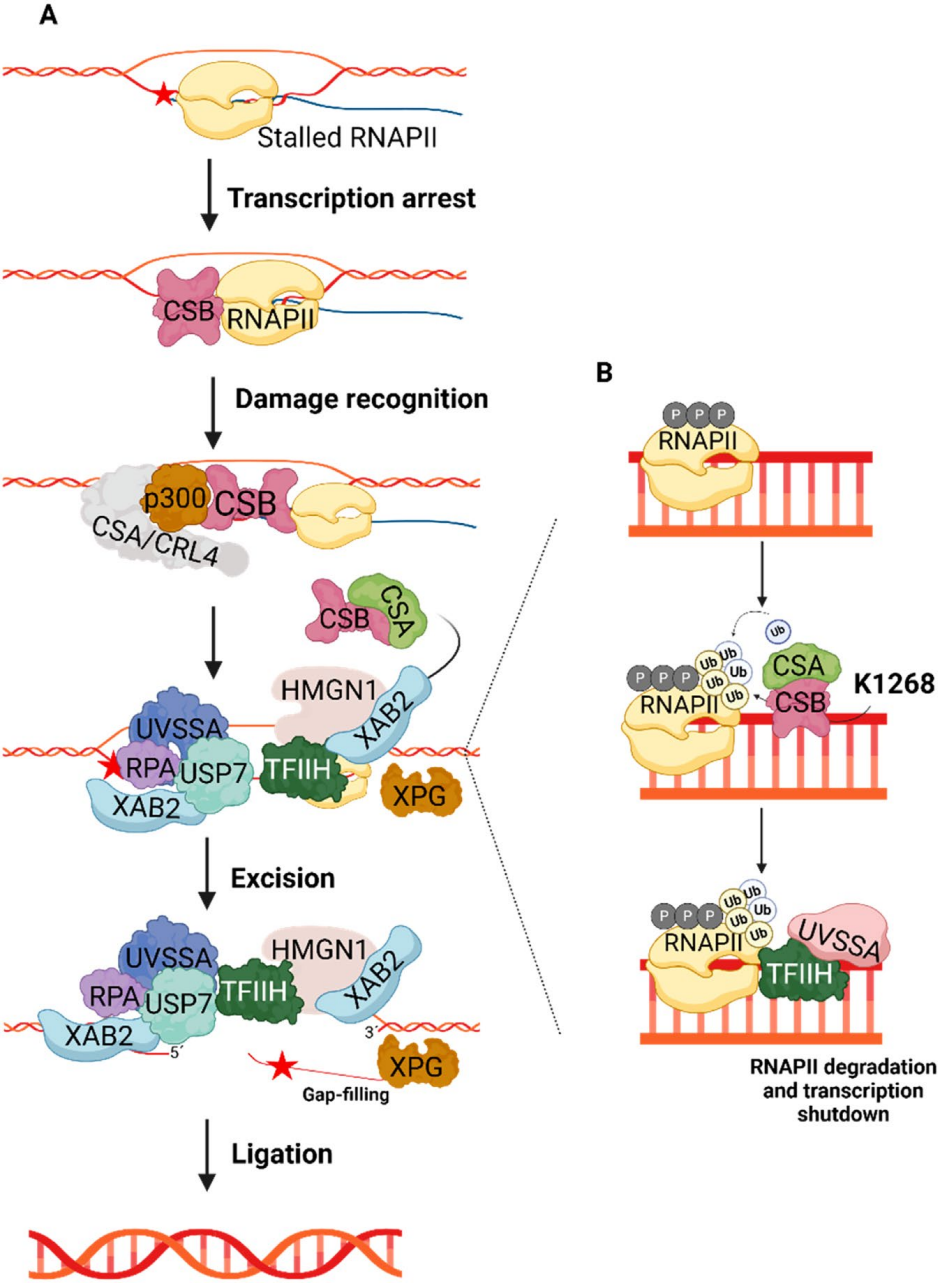
Schematic model of ERCC6 role in TC‐NER. (A) Helix‐distorting lesions (red star) lead to the stall of RNA polymerase II (RNAPII) which causes transcription arrest and ERCC6 recruitment upon damage recognition. ERCC6 then alters its conformation and recruits the ERCC8/cullin 4‐RING ubiquitin ligase (CRL4) complex and the histone acetyltransferase p300 to the arrest site. Posteriorly, ERCC6 also recruits nucleotide excision repair (NER) factors, Replication protein A RPA, ERCC5 and Transcription factor IIH (TFIIH), and transcription coupled nucleotide excision repair (TC‐NER) regulatory proteins, UVstimulated scaffold protein A (UVSSA), Ubiquitin carboxyl‐terminal hydrolase 7 (USP7) and XPA‐binding protein 2 (XAB2). In addition, aided by ERCC8, ERCC6 recruits the chromatin remodeler, High mobility group nucleosome‐binding domain‐containing protein 1 (HMGN1) to the lesion. Following lesion recognition, the recruited factors induce lesion excision and nick is sealed by ligase upon gap‐filling. (B) During (TC‐NER), ERCC6 forms a complex with ERCC8 and is recruited to the stalled RNAPII. This process leads to RNAPII ubiquitination at residue K1268 facilitating UVstimulated scaffold protein A (UVSSA) and Transcription factor II H (TFIIH) interaction with the stalled RNAPII. Following RPB1 (a RNAPII subunit)‐K1268 ubiquitination, the stalled RNAPII is processed and degraded, culminating in transcription shutdown. Created with BioRender.com.

CS‐B is associated with an impairment in TC‐NER, considering ERCC6 is pivotal in this pathway, representing the most studied extensively researcher molecular function of ERCC6 (Figure 2A). This section will focus solely on the role of ERCC6 in TC‐NER, as the complete molecular pathway has been detailed elsewhere (Duan et al. 2021). In TC‐NER, Helix‐distorting lesions such as UV‐induced DNA damage in actively transcribed genes result in stalling of RNAPII, thereby hindering the access of DNA repair enzymes to the damaged region (Hanawalt and Spivak 2008). Following RNAPII arrest, ERCC6 is recruited to the lesion site by RNAPII, resulting in the formation of the ERCC6‐RNAPII complex, which marks the initiation of TC‐NER (Tiwari et al. 2021). ERCC6 then alters its conformation through an interplay between the ATPase domain and the N and C terminals, thereby revealing the DNA binding domain and enabling it to wrap around the DNA (Batenburg et al. 2018; Lake et al. 2010). This event is followed by the ERCC6‐mediated recruitment of the ERCC8/E3 ubiquitin ligase complex (CRL4) and histone acetyltransferase p300 to the arrest site (Duan et al. 2021). Furthermore, ERCC6 is also responsible for the recruitment to the lesion site of the core NER factors, including Replication protein A (RPA), ERCC5 and transcription factor IIH (TFIIH) complex, as well as TC‐NER specific regulatory proteins, such as UVSSA, Ubiquitin carboxyl‐terminal hydrolase 7 (USP7) and XPA‐binding protein 2 (XAB2) (Fousteri and Mullenders 2008; Tiwari et al. 2021). Finally, alongside ERCC8, ERCC6 recruits the chromatin remodeler, High mobility group nucleosome‐binding domain‐containing protein 1 (HMGN1) to the stalled RNAPII (Aamann et al. 2013). These ERCC6‐recruited components collectively aid the recognition and excision of the lesion. Subsequently, the lesion is removed, the created gap filled by DNA polymerase, and each end ligated by a ligase.

Beside recruiting several factors during TC‐NER, ERCC6 also modulates RNAPII ubiquitination and thereby its processing fate (Figure 2A) (He, Zhu, et al. 2017). During TC‐NER, the complex formed by ERCC6 and ERCC8/CRL4, aided by elongation factor 1 (ELOF1), participates in the K1268 ubiquitination of RPB1, a RNAPII subunit (Anindya et al. 2007; Nakazawa et al. 2020; Nakazawa et al. 2012; van der Weegen et al. 2021). This specific ubiquitination regulates stalled RNAPII processing and degradation, which is important for transcription shutdown and posterior UV damage recovery response (Nakazawa et al. 2020; Tufegdžić Vidaković et al. 2020). Moreover, RPB1‐K1268 ubiquitination promotes the association of UVSSA and TFIIH with stalled RNAPII, both of which are key factors in TC‐NER initiation (Nakazawa et al. 2020; Nakazawa et al. 2012). Interestingly, the mechanism by which ERCC6 regulates RNAPII levels remains elusive, considering contradictory findings regarding whether ERCC6 promotes (Nakazawa et al. 2020) or inhibits (Tufegdžić Vidaković et al. 2020) RNAPII degradation.

#### 2.1.2 Interstrand Crosslink (ICL) Repair

DNA interstrand crosslinks are lesions characterized by covalent bonds between antiparallel DNA strands, caused by lipid peroxidation or chemotherapeutic agents. These lesions prevent DNA replication and block transcription machinery assembly, making them extremely cytotoxic (Enoiu et al. 2012). The removal of some ICL types is dependent on functional NER. Therefore, increasing evidence suggests ICL repair requires ERCC6 and relies on TC‐NER in a replication‐independent manner (Enoiu et al. 2012; Furuta et al. 2002; Iyama and Wilson 2016). Additionally, ERCC6 is swiftly recruited and accumulates at ICL sites. Further reinforcing the significance of ERCC6 in ICL repair, ERCC6 interacts and colocalizes with nitrogen mustard 1A (SNM1A), a 5′–3′ exonuclease involved in ICL repair, at ICL sites. Moreover, ERCC6 enhances SNM1A catalytic activity and promotes its recruitment to DNA damage sites (Iyama et al. 2015). This collective evidence, although not conclusive, points to an important role of ERCC6 in ICL repair.

#### 2.1.3 DNA Base Excision Repair (BER)

**FIGURE 3:**
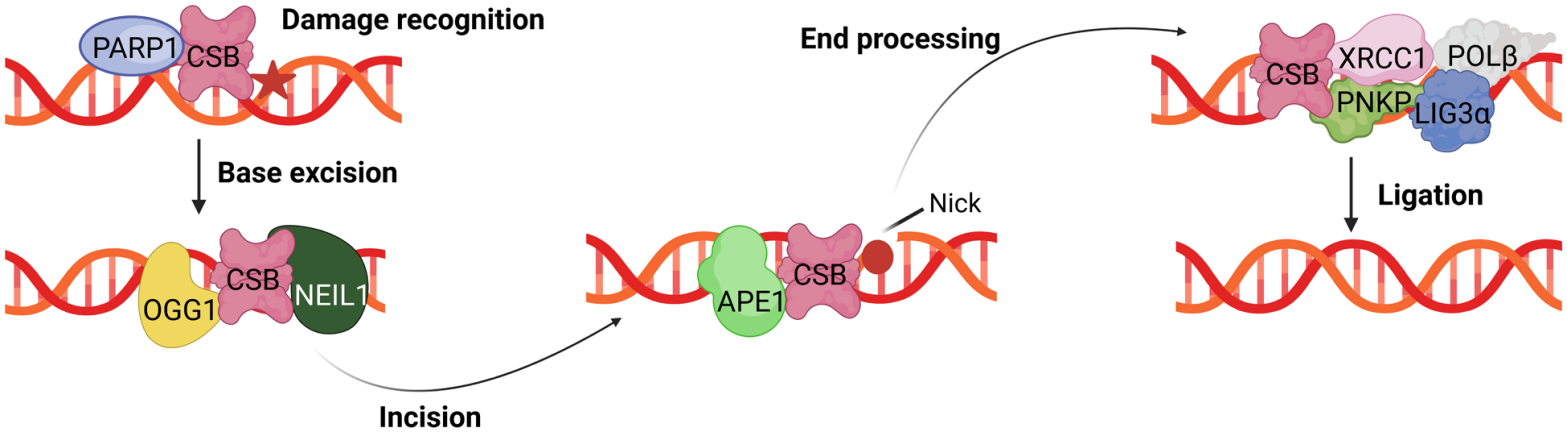
Schematic model of ERCC6 involvement in BER. Nuclear base excision repair (BER) is initiated by the interaction of ERCC6 and poly(ADP‐ribose)polymerase (PARP‐1) which leads to the DNA oxidative lesion (red star) recognition. ERCC6 then stimulates factors 8‐Oxoguanine glycosylase (OGG1) function and physically interacts with Nei Like DNA Glycosylase 1 (NEIL1) leading to damaged base removal (red circle). ERCC6 interacts with Apurinic/apyrimidinic endonuclease 1 (APE1) inducing an incision. Following that, ERCC6 stimulates X‐ray repair cross‐complementing protein 1 (XRCC1) promoting the recruitment of polynucleotide Kinase 3’‐Phosphatase (PNKP), DNA polymerase β (POLβ), and DNA ligase IIIa (LIG3α) culminating in gap ligation. Created with BioRender.com.

ERCC6 is additionally involved in other DNA repair mechanisms, although these are less thoroughly described than its function in TC‐NER. One of these mechanisms, BER, is responsible for repairing small non‐helix‐distorting lesions that primarily result from oxidative DNA damage. This process occurs in the nucleus and mitochondria; yet, regardless of the final localization, BER factors are encoded by nuclear genes (Tiwari et al. 2021). ERCC6 has been described as interacting physically and/or functionally with several important BER factors (Figure 3). In the nucleus, ERCC6 stimulates the function of 8‐Oxoguanine glycosylase (OGG1) (Tuo et al. 2002) and physically interacts with Nei Like DNA Glycosylase 1 (NEIL1) (Muftuoglu et al. 2009) and Apurinic/apyrimidinic endonuclease 1 (APE1) (Wong et al. 2007), in turn increasing their activity. Additionally, poly(ADP‐ribose)polymerase (PARP‐1) and ERCC6 form a complex that recognizes and binds to DNA oxidative lesions, thereby promoting BER (Boetefuer, Lake, Dreval, et al. 2018; Flohr et al. 2003). Beyond its role concerning BER effector proteins, ERCC6 stimulates X‐ray repair cross‐complementing protein 1 (XRCC1), a non‐enzymatic scaffold protein in BER that binds to single‐stranded DNA breaks. Posteriorly, XRCC1 recruits other additional proteins, including Polynucleotide kinase 3′‐phosphatase (PNKP), DNA polymerase‐β (POLβ), and DNA ligase IIIα (LIG3α) to finalize BER (Menoni et al. 2018).

#### 2.1.4 DNA Double‐Strand Break Repair

**FIGURE 4:**
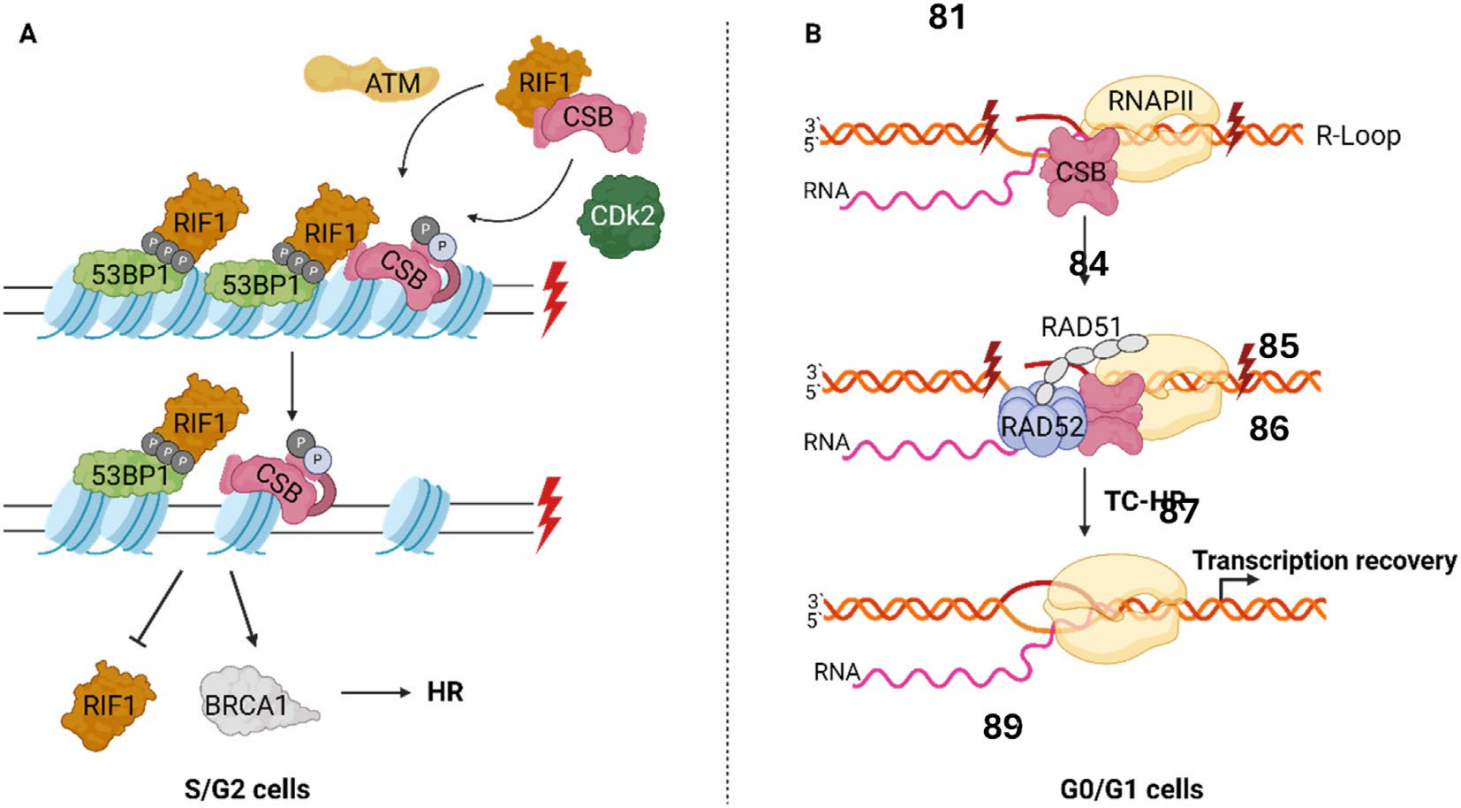
Schematic for ERCC6‐mediated DSB repair pathway choice. (A) Following ionizing radiation (IR)‐induced damage, ERCC6 is phosphorylated by ataxia telangiectasia mutated (ATM) and cyclin‐dependent kinase 2 (CDK2), facilitating chromatin remodeling at double‐strand break (DSB) sites. This process leads to the repression of regulatory factor 1 (RIF1) and P53 Binding Protein 1 (53BP1) while promoting breast cancer 1 (BRCA1)‐mediated Homology Recombination (HR), during the S/G2 cell cycle phase. (B) Reactive oxygen species (ROS) induce the formation of DSBs at transcribed regions, leading to R‐loop formation. ERCC6 detects/binds to the R‐Loop and interacts with RAD52, facilitating its recruitment. ERCC6‐RAD52 promotes RAD51 loading at R‐loops enabling transcription‐coupled homologous recombination (TC‐HR), during the G0/G1 cell cycle phase. Created with BioRender.com.

Double stranded breaks DNA breaks may originate through several processes, including direct damage from ionizing radiation, or endogenous events such as DNA processing and replication (Tiwari et al. 2021). DSBs are mainly repaired by homologous recombination (HR), in the presence of a template DNA and during S/G2 cell cycle phase, or alternatively through nonhomologous end joining (NHEJ) in the absence of template DNA (Batenburg et al. 2019). ERCC6 plays a role in determining which of these pathways are responsible for repairing DSBs. This process is initiated following the removal of histones surrounding the lesion by ERCC6 in an Ataxia telangiectasia mutated (ATM) and Cyclin‐dependent kinase 2 (CDK2) controlled manner. Through this remodulation, ERCC6 promotes Breast cancer 1 (BRCA1)‐mediated homology recombination repair, while repressing replication timing regulatory factor 1 (RIF1) and P53 Binding Protein 1 (53BP1) mediated NHEJ (Figure 4A) (Batenburg et al. 2019; Batenburg et al. 2017). Moreover, ERCC6 has also been demonstrated to participate in the maintenance of DNA damage checkpoints, following DSBs formation (Batenburg et al. 2015).

Interestingly, ERCC6 has also been found to mediate DSB repair at transcriptional active sites through transcription‐coupled homologous recombination (TC‐HR) during G0/G1 cell cycle phase (Wei et al. 2015). This mechanism, in contrast to ERCC6‐mediated HR during S/G2, is independent of BRCA1/2 and utilizes RNA as a repair template rather than sister chromatids, which are unavailable during this cell cycle phase (Teng et al. 2018). In TC‐HR is proposed that DSBs may lead to the formation of R‐loops, which are DNA:RNA hybrids formed when template DNA hybridizes with a nascent RNA molecule during transcription. ERCC6 detects and binds to the newly formed R‐loop, facilitating its interaction with RAD52 and its recruitment to damage site (Figure 4B). Finally, ERCC6‐RAD52 promotes RAD51 recruitment, leading to homologous recombination and transcriptional recovery (Sollier et al. 2014; Teng et al. 2018; Wei et al. 2015).

### 2.2 ERCC6‐Mediated Transcription Modulation

#### 2.2.1 Chromatin Remodeling

ERCC6 has been extensively described for its role in chromatin remodeling through its ATPase domain, which is essential for ERCC6‐mediated transcription and DNA repair (Citterio et al. 2000; Newman et al. 2006). Notably, homodimerization of ERCC6 mediated by the ATPase domain is required for its chromatin remodeling activity (Christiansen et al. 2005). In addition, the ATPase domain mediates DNA wrapping and unwrapping of ERCC6 around the DNA through ATP binding or hydrolysis, respectively. Ultimately, this process allows ERCC6 to actively alter the conformation of the DNA double helix, therefore influencing the interaction of DNA with nucleosomes and other proteins (Beerens et al. 2005; Muftuoglu et al. 2006). In accordance with the function of other chromatin remodeling factors, ERCC6 is suggested to solely regulate a specific subset of genes (Boetefuer, Lake, Dreval, et al. 2018; Newman et al. 2006).

The ATPase activity of ERCC6 is regulated by post‐translational modifications. In line with this, ERCC6 dephosphorylation, following UV exposure, enhances ERCC6 ATPase activity (Christiansen et al. 2003). Contrariwise, ERCC6 phosphorylation by Abelson murine leukemia viral oncogene homolog 1 (ABL1) or PARylation by PARP1 in response to oxidative stress negatively regulates ERCC6 ATPase activity, therefore modulating ERCC6 chromatin remodeling activity (Imam et al. 2007; Thorslund et al. 2005). Notably, PARP1 promotes ATPase‐independent association of ERCC6 to the chromatin under oxidative stress conditions; nevertheless, the ERCC6 ATPase domain remains essential in cooperation with the ERCC6 C‐terminal to stabilize the ERCC6‐chromatin association (Boetefuer, Lake, Dreval, et al. 2018). Furthermore, the association of ERCC6 with chromatin following UV exposure requires active RNAPII transcription, whereas during oxidative stress, active transcription is mostly dispensable, indicating that the mechanism underlying ERCC6‐chromatin association induced by UV or oxidative stress is distinct (Bilkis et al. 2023; Boetefuer, Lake, Dreval, et al. 2018). Nevertheless, due to the complexity of these mechanisms, additional research is required to elucidate ERCC6 chromatin association in response to different stressors.

#### 2.2.2 Transcription Regulation

**FIGURE 5:**
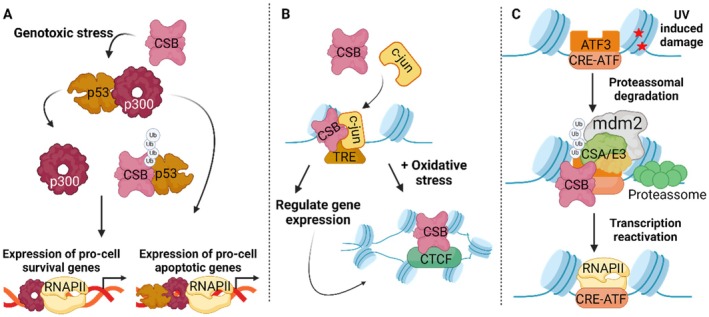
Model for transcription regulation by ERCC6 under different conditions. (A) Following genotoxic stress, ERCC6 physically interacts with P53 preventing its interaction with the histone acetyltransferase p300. This process prompts pro‐survival gene expression instead of pro‐apoptotic gene expression. (B) Under physiological conditions, during the transcription initiation stage, ERCC6 has a high occupancy at 12‐O‐tetradecanoylphorbol‐13‐acetate (TPA) response elements (TREs) modulated by the transcription factor c‐jun, which prompts ERCC6 regulation of specific gene expression. This process also occurs under oxidative stress conditions with the addition of ERCC6 altering its genomic localization and interacting with CCCTC‐binding factor (CTCF). This interaction leads to mutual chromatin regulation and, consequently, gene expression regulation. (C) Upon UV damage, ERCC6 assisted by ERCC8/E3 ubiquitin ligase complex and murine doble minute 2 (mdm2), promotes transcription factor 3 (ATF3) ubiquitin‐mediated proteasomal degradation. RNAPII is recruited and ATF3‐responsive genes transcription is reactivated. Created with BioRender.com.

The ATPase domain of ERCC6 is crucial for transcriptional regulation, regardless of the presence or absence of DNA damage. ERCC6 regulatory function is particularly critical in response to seemingly global transcription impairment caused by oxidative stress (Kyng et al. 2003). Following oxidative stress, in addition to transcription, ERCC6 not only promotes transcription but also regulates upstream regulation of stress responsive genes, as well as genes associated with translation and cell cycle (Kyng et al. 2003). Consequently, stimulated by exacerbated oxidative conditions, ERCC6 alters its genomic localization, particularly increasing occupancy at target promoter sites (Lake et al. 2016). Moreover, oxidative stress promotes the direct interaction between ERCC6 and the long‐range chromatin‐structure regulator, CCCTC‐binding factor (CTCF), which regulates ERCC6 occupancy at specific genomic loci (Figure 5B) (Lake et al. 2016; Lee and Iyer 2012). Likewise, ERCC6‐CTCF interaction regulates the association of CTCF with chromatin under conditions of oxidative stress. Ultimately, ERCC6 and CTCF regulate each other's chromatin association, leading to a coordinated gene expression regulation in response to oxidative stress (Gray et al. 2012; Lake et al. 2016).

Upon UV‐induced damage, ERCC6, in combination with ERCC8/E3 ubiquitin ligase complex and Murine double minute 2 (Mdm2), promotes ubiquitin‐mediated proteasomal degradation of activating transcription factor 3 (ATF3), a transcriptional repressor that recognizes CRE/ATF‐binding sites (Figure 5C). After ATF3 degradation, RNAPII is recruited, leading to the restoration of transcription for ATF3‐responsive genes (Epanchintsev et al. 2017; Kristensen et al. 2013). Notably, ERCC6‐promoted degradation of ATF3 may not constitute the main mechanism underlying transcription recovery upon UV damage, considering RNAPII processing has also been implicated in transcription recovery.

Beyond regulating transcription machinery, ERCC6 is suggested to be directly involved in transcription initiation, considering its strong correlation to regions exhibiting epigenetic features of promoter and enhancer regions. In line with this, ERCC6 occupancy at 12‐O‐tetradecanoylphorbol‐13‐acetate (TPA) response elements (TREs) is enriched. Moreover, ERCC6 occupancy at TREs is suggested to be modulated by the sequence‐specific transcription factor c‐Jun, which can be found in the same protein‐DNA complex as ERCC6 (Figure 5B). Consequently, c‐Jun‐mediated recruitment of ERCC6 prompts ERCC6 regulation of gene expression in specific genomic regions during the transcription initiation stage (Lake et al. 2014).

#### 2.2.3 rDNA Transcription

ERCC6 has been described to localize to the nucleolus, the site of rDNA transcription. Furthermore, ERCC6 integrates a complex comprised of RNAPI, TFIIH, ERCC5, and transcription termination factor 1 (TTF‐1), which is important in RNAPI transcription of rDNA (Bradsher et al. 2002; Iyama et al. 2018).

**FIGURE 6:**
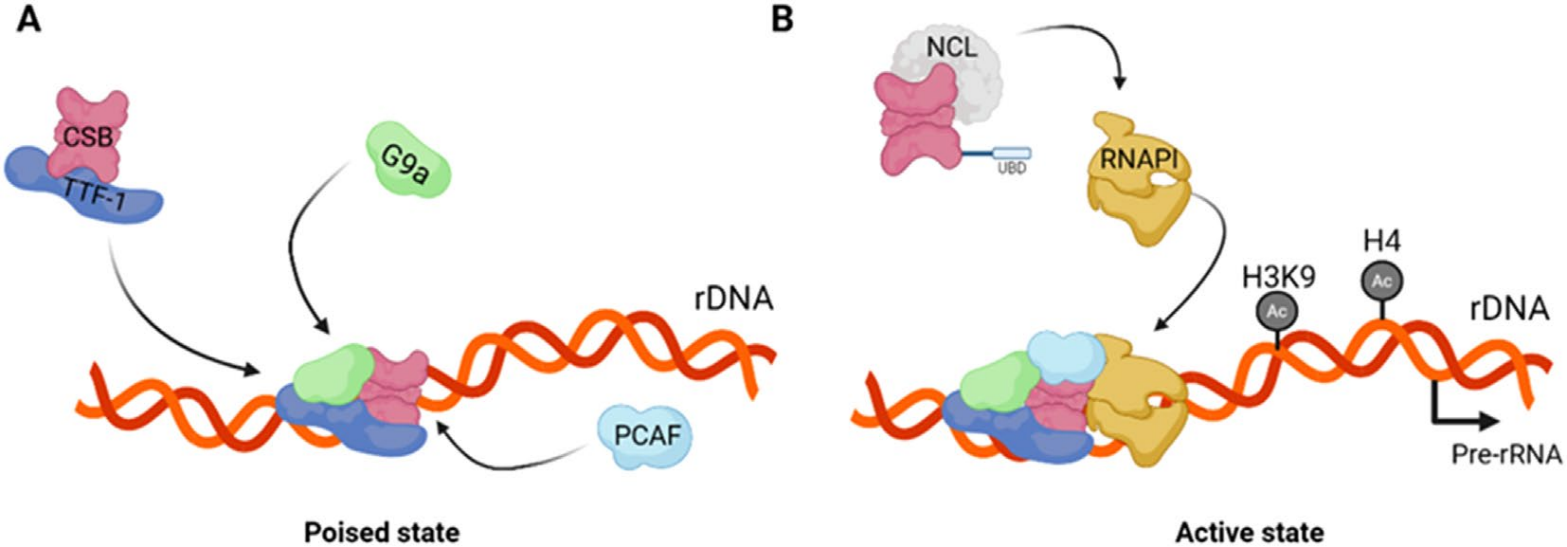
Model for the role of ERCC6 in rDNA transcription. (A) ERCC6 and transcription termination factor 1 (TTF‐1) form a complex that facilitates the methyl transferase G9a recruitment to ribosomal DNA (rDNA) in a poised state. The histone acetyltransferase PCAF is recruited by ERCC6 leading to histone 3K9 (H3K9) and histone 4 (H4) acetylation enabling the transcription initiation complex assembly. (B) This process induces the transition from poised rDNA to an active state. ERCC6´s interaction with nucleolin (NCL) enhances RNAPI loading to the rDNA coding region facilitating rRNA transcription regulation. Created with BioRender.com.

Notably, ERCC6 regulates chromatin remodeling and epigenetic modifications both of which are involved in the modulation of rRNA transcription (McStay and Grummt 2008). Consistently, ERCC6 and TTF‐1, which directly interact, facilitate RNAPI‐mediated transcription by recruiting chromatin remodelers (Längst et al. 1998; Yuan et al. 2007). It is though that the direct interaction between ERCC6 and G9a, a methyl transferase involved in RNAPI transcription regulation, promotes the recruitment of G9a to rDNA following the complexation of TFF‐1 and ERCC6 (Figure 6A). Through this interaction, G9a modulates histone 3 lysine 9 (H3K9) methylation, thereby prompting rDNA transcription (Yuan et al. 2007). ERCC6 role in promoting epigenetic regulation of rDNA transcription is further reinforced by the recruitment of p300/CBP‐associated factor (PCAF), a histone acetyltransferase, to poised rDNA by ERCC6. Subsequently, histone 4 (H4) and H3K9 acetylation by PCAF, promotes the assembly of the transcription initiation complex, transitioning poised rDNA into a transcriptionally active state (Figure 6A) (Shen et al. 2013). Besides indirectly promoting histone modification that culminate in rRNA synthesis, ERCC6 interacts with Nucleolin (Ncl), a major nucleolar protein, involved in rDNA transcription, pre‐rRNA processing and ribosomal assembly (Figure 6B). In fact, ERCC6 enhances RNAPI loading into the rDNA coding region in a Nucleolin‐dependent manner, suggesting that the coordination between ERCC6 and Ncl regulates rRNA transcription and ribosomal biogenesis (Okur, Lee, et al. 2020). Finally, ERCC6 utilizes its helicase activity to dismantle the abundant secondary structures in rDNA known as G‐quadruplexes (G4), in a transcription‐dependent manner, which minimizes transcriptional stalling at G4 structure sites (Scheibye‐Knudsen et al. 2016).

#### 2.2.4 Cell Fate

Cell fate is tightly regulated by finely‐tuned networks that determine whether cells divide, arrest cell cycle or initiate cell death programs (Tatapudy et al. 2017). The transcriptional master regulator, p53, is a crucial factor in the regulation of cellular responses to genotoxic stress, including cell cycle arrest, apoptosis and cell senescence (Paccosi and Proietti‐De‐Santis 2021). ERCC6 interacts with p53 and assisted by ERCC8 promotes its ubiquitination and subsequent degradation, preventing p53 interaction with p300 (Figure 5A) (Latini et al. 2011; Wang et al. 1995). Thus, ERCC6 competes for p53 binding with p300, prompting expression of pro‐cell survival genes that promote cell proliferation and survival. Conversely, in the case p300 is bound to p53, a pro‐apoptotic transcriptional response that leads to cell cycle arrest and cell death (Filippi et al. 2008; Frontini and Proietti‐De‐Santis 2009; Latini et al. 2011). Additionally, ERCC6 through its interaction with p53, an antagonist of HIF‐1 pathways, prevents p53 from attenuating HIF‐1 controlled response during hypoxia and promotes cell survival (Filippi et al. 2008). Notably, p53 and ERCC6 are engaged in a negative feedback loop, where p53 binds to the *ERCC6* gene promoter and transcriptionally controls *ERCC6* expression. Once ERCC6 is upregulated p53 returns to baseline levels (Frontini and Proietti‐De‐Santis 2012; Latini et al. 2011). In a p53‐indenpent manner, ERCC6 prevents cell senescence following cytotoxic stress by regulating p21 expression. In fact, ERCC6 binds to the p21 promoter and downregulates its transcription countering p21‐triggered cell senescence. Interestingly, CSB promoter H3K5 hypoacetylation is a marker of replicative senescence (Crochemore et al. 2019). Finally, ERCC6 has also been found to be implicated in cell cycle regulation. During cytokinesis, ERCC6 associated with ERCC8 localizes to the midbody and promotes the ubiquitination/degradation of PRC1, a key component of intercellular bridges (Paccosi et al. 2020).

### 2.3 Mitochondrial Homeostasis

**FIGURE 7:**
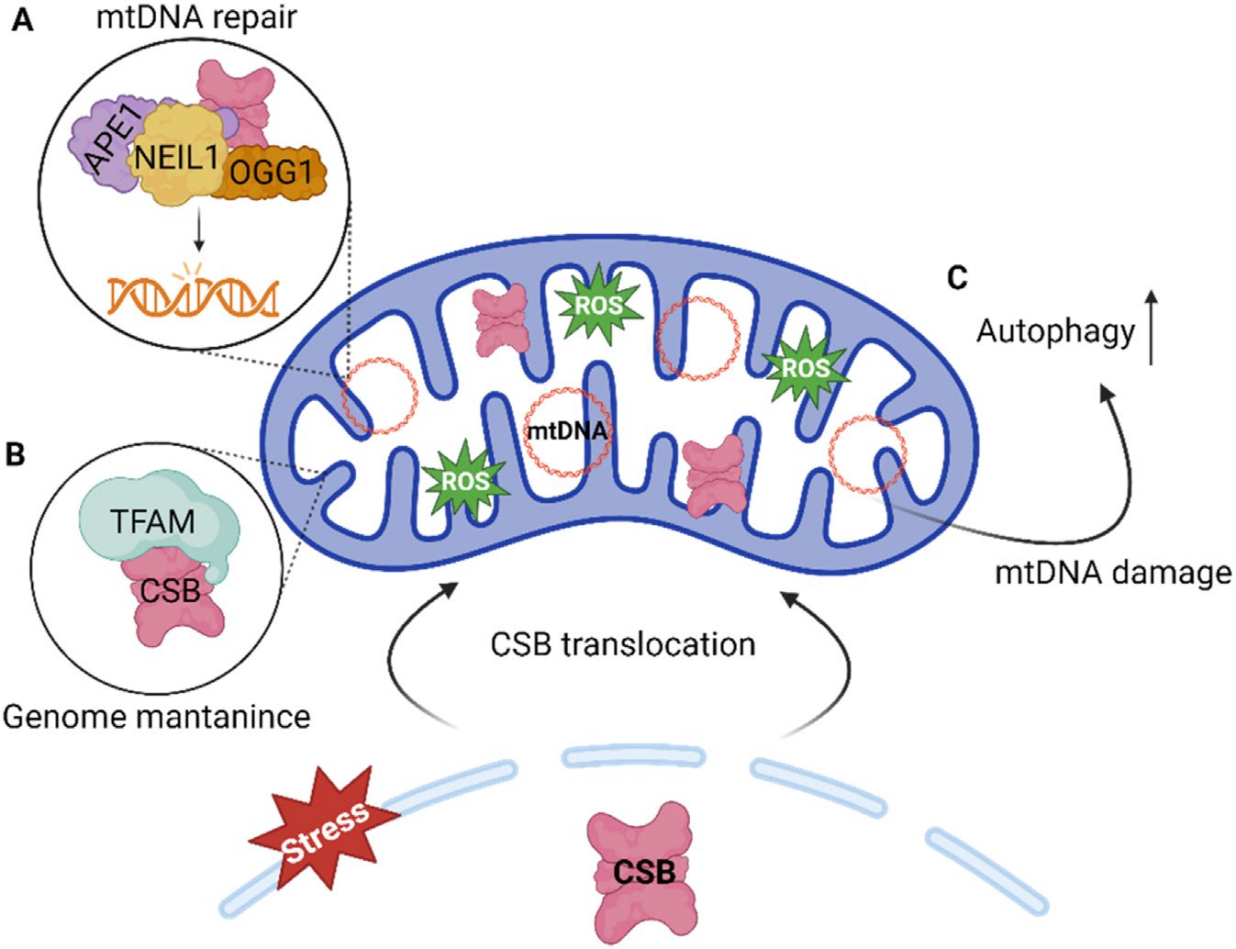
Model for ERCC6 role in mitochondrial homeostasis. (A) In oxidative stress conditions, ERCC6 translocates from the nucleus to the mitochondria where it modulates the base excision repair (BER) factors 8‐Oxoguanine glycosylase (OGG1), Nei Like DNA Glycosylase 1 (NEIL1) and apurinic/apyrimidinic endonuclease 1 (APE1) contributing to mitochondrial DNA (mtDNA) damage repair. (B) In the mitochondria nucleoid, ERCC6 interacts with mitochondrial transcription factor A (TFAM) prompting mitochondrial transcription. (C) Additionally, in response to oxidative stress, ERCC6 promotes mitochondrial autophagy. Created with BioRender.com.

The role of ERCC6 in mitochondria is still not fully understood, however, mitochondrial dysfunction and related features have been extensively described in CS cell and animal models (Pascucci et al. 2012; Scheibye‐Knudsen et al. 2012). These features range from altered redox balance, cellular bioenergetics changes, apoptosis‐mediated loss of subcutaneous fat, stress‐induced mitochondrial DNA (mtDNA) damage, defective mitochondrial transcription (Aamann et al. 2013; Berquist et al. 2012; Kamenisch et al. 2010; Osenbroch et al. 2009; Pascucci et al. 2012). In line with these observations, ERCC6 was found to translocate to mitochondria in response to oxidative stress (Figure 7). There, ERCC6 is thought to be involved in mtDNA repair by modulating the activity of BER factors, including OGG1, NEIL1, and APE, which are also present in the mitochondria (Figure 7A) (Aamann et al. 2010; Kamenisch et al. 2010; Muftuoglu et al. 2009). Moreover, an organizational role for ERCC6 in the mitochondria nucleoid has also been suggested, attributed to its interaction with Mitochondrial transcription factor A (TFAM), a protein widely involved in mitochondrial genomic processes (Figure 7B) (Berquist et al. 2012; Kamenisch et al. 2010). This idea is further reinforced by the discovery that ERCC6 promotes mitochondrial transcription (Berquist et al. 2012). Additionally, ERCC6 is suggested to act as a genotoxic sensor, triggering mitochondrial autophagy in response to mtDNA damage (Figure 7C) (Scheibye‐Knudsen et al. 2012). Interestingly, ERCC6 interacts with the mitochondrial protein 3‐hydroxyisobutyrylcoenzyme, an important hydrolase (HIB‐CoA) in the amino acid metabolism. The function of this interaction remains unclear; however, it has been suggested to be involved in mitochondrial nucleoid structure (Aamann et al. 2010). This reveals a potential ERCC6 role beyond DNA‐associated functions, however, this interaction has not been described in mammalian cells.

## 3 CS‐B Pathogenesis

Aging entails a progressive loss of cellular integrity resulting from cumulative damage and impaired cellular functions, ultimately leading to increased susceptibility to death (López‐Otín et al. 2013, 2023). Most hallmarks of aging are also present in CS‐B, including genomic instability, epigenetic alterations, loss of proteostasis, and mitochondrial dysfunction. These similarities highlight the premature aging observed in patients with this ultra rare and highly debilitating disorder (López‐Otín et al. 2013; Tiwari et al. 2021).

Despite the parallels with aging, CS pathogenesis remains poorly elucidated and subject to disagreement in the field. It is uncertain whether CS‐B pathogenesis has a primary disease‐causing mechanism or if its pathogenesis arises from a combination of multiple mechanisms, which in isolation do not produce a phenotype. Numerous hypotheses concerning the disease mechanisms underlying CS‐B pathogenesis have been proposed throughout the years. The CS‐B phenotype can be reproduced by specific mutations in genes involved in TC‐NER, other than *ERCC6*. These TC‐NER components capable of replicating the CS‐B phenotype upon mutation probably lack the same non‐TC‐NER functions of ERCC6. Therefore, it has been proposed that CS‐B does not result from dysfunctions in other ERCC6 functions but rather from defects associated with TC‐NER (Lans et al. 2019). Notably, it has been suggested that CS‐B pathogenesis is linked to impaired processing of stalled RNAPII, which prevents DNA repair machinery from accessing transcription blocking lesions, rather than failure to remove DNA lesions (Jia et al. 2021; van den Heuvel et al. 2021).

Many clinical manifestations of CS‐B overlap with those observed in mitochondrial disorders. Furthermore, mitochondrial dysfunction has been extensively documented in CS‐B, even in the absence of DNA damage (Chatre et al. 2015; Scheibye‐Knudsen et al. 2013). In line with this, ERCC6‐deficient cells show altered energy metabolism, accumulation of mtDNA lesions, elevated reactive oxygen species (ROS) production, and accumulation of damaged mitochondria, all of which are hallmarks of mitochondrial dysfunction. Based on these findings, several studies have suggested that mitochondrial dysfunction is a key contributor to CS‐B pathogenesis that directly contributes to the progeroid phenotype found in CS‐B (Chatre et al. 2015; Kamenisch and Berneburg 2013; Okur, Fang, et al. 2020; Scheibye‐Knudsen et al. 2013).

Based on findings from cell and animal models, it has been proposed that CS‐B arises from a combination of altered gene transcription, impaired DNA repair mechanisms, and mitochondrial dysfunction (Vessoni et al. 2020). Furthermore, an integrative model elucidating the diverse functions of ERCC6 posits that CS‐B arises from defects in ubiquitin‐mediated degradation mechanisms dependent on ERCC6. This model integrates the functions of ERCC6 in RNAPII processing, p53 regulation, ATF3 degradation, cytokinesis, and ribosomal metabolism (Costanzo et al. 2024; Paccosi and Proietti‐De‐Santis 2021).

Despite the nervous system protection against UV‐induced DNA damage, it remains the most severely affected tissue in CS‐B. This may be associated with functions of ERCC6 beyond TC‐NER, which is conventionally tied to UV‐damage repair. Neurons exhibit high transcriptional activity and depend on error‐free gene expression, rendering them vulnerable to transcriptional stalling and endogenous DNA damage (Wilson 3rd et al. 2023). Furthermore, neurons are significantly dependent on mitochondrial energy metabolism, making them highly susceptible to energy deficits resulting from mitochondrial dysfunction (Cunnane et al. 2020). Taking this into consideration, we propose that the pathogenesis of CS‐B arises from a combination of three main molecular pathological features: (i) DNA damage accumulation, (ii) transcriptional dysregulation, and (iii) mitochondrial dysfunction. Furthermore, considering potential interactions between these three CS‐B molecular features, we hypothesize that CS‐B pathogenesis cannot be explained by one pathogenic mechanism or a combination of independent disease mechanisms. Rather, it stems from an interaction of interconnected pathways that may exacerbate one another and perpetuate cellular dysfunction.

- DNA damage accumulation

Accumulation of DNA damage, such as mutations, aberrant DNA structures and stalled transcription, can impair DNA replication or compromise the function or expression of the affected genomic regions (Schumacher et al. 2021). ERCC6 plays important functions in several DNA repair pathways that respond to different types of damaging agents. Thus, the absence or functional deficiency of ERCC6 will result in dysfunctional DNA repair mechanisms and sensitivity to DNA damaging agents. In line with this notion, ERCC6‐deficient cells display impairments in the recruitment of DNA repair machinery of TC‐NER (Fousteri and Mullenders 2008), BER (Menoni et al. 2018), and DSB repair (Batenburg et al. 2019), while also presenting dysfunctional modulation of specific BER (Wong et al. 2007) and ICL repair (Iyama et al. 2015) factors. Hence, it is expected that ERCC6‐deficient cells will be unable to cope with DNA damage due to impaired ERCC6‐dependent recruitment or activity regulation of DNA repair factors. This will result in transcriptional stress, caused by the continuous accumulation of DNA abnormalities that reduce transcription rate, compromise transcription fidelity, or completely halt transcription (Kajitani et al. 2021; Lans et al. 2019). Consequently, DNA‐damage induced transcriptional stress triggers aging‐related gene expression profiles (Gyenis et al. 2023). The inability to remove ATF3 from the DNA is hypothesized to hinder the accessibility of DNA repair machinery, leading to the accumulation of DNA damage in ATF3 responsive genes (Epanchintsev et al. 2017; Paccosi and Proietti‐De‐Santis 2021). On the other hand, alterations in coding and non‐coding DNA genomic regions may lead to changes in the protein sequences and regulatory non‐coding RNAs such as miRNA, impairing their physiological function. Ultimately, the progressive accumulation of defects in DNA will become unbearable and instate a general state of cellular dysfunction.

ii Transcriptional dysregulation

Transcription dysregulation occurs when regulatory DNA sequences are disrupted by mutations or impaired regulatory elements, including transcription factors, transcription co‐factors and chromatin remodelers (Lee and Young 2013). This dysregulation may lead to the transcription repression of genes essential for cellular homeostasis, or unregulated activation of genes that adversely affect the cell (Latini et al. 2011). The regulatory function of ERCC6 in transcription is crucial in response to genotoxic stress, such as oxidative and UV damage. Accordingly, ERCC6‐deficient cells exhibit many differentially regulated genes associated with DNA repair, ribosomal functions, and signal transduction, in response to genotoxic stress, hindering the cell's ability to promote an adaptive gene expression program (Boetefuer, Lake and Fan 2018; Kyng et al. 2003). Consequently, ERCC6 patients display downregulation of genes related to cell cycle regulation and altered signal transduction as well as protein turnover upon oxidative damage (Kyng et al. 2003). Dysfunctional ERCC6, while under different cellular stress conditions, fails to interact with p53, leading to upregulation of ERCC6 and activation of pro‐apoptotic gene expression (Filippi et al. 2008; Frontini and Proietti‐De‐Santis 2009, 2012; Latini et al. 2011). Defects in ERCC6‐mediated modulation of RNAP I and II disrupt transcription, potentially affecting the expression of regions adjacent to ERCC6 occupied sites (Alupei et al. 2018; Lake et al. 2014). *ERCC6* mutations may compromise its interaction with CTCF, impairing ERCC6's ability to respond to oxidative stress in a CTCF‐dependent manner (Lake et al. 2016). Likewise, following UV‐induced damage, dysfunctional ERCC6 is incapable of promoting the degradation of ATF3, disrupting subsequent RNAPII recruitment and blocking transcription of ATF3 responsive genes (Epanchintsev et al. 2017; Kristensen et al. 2013). Ultimately, transcription dysfunction will promote a pathological expression profile that impairs the cellular ability to respond to genotoxic stimuli and will also disrupt finely tuned cell survival checkpoints.

iii Mitochondrial dysfunction

Mitochondrial dysfunction is underlined by mitochondrial DNA (mtDNA) damage, impairments in the mitochondrial electron transport chain (ETC) and oxidative stress, all of which, once instated further exacerbate one another (Li et al. 2024). ERCC6 has important roles in the maintenance of mtDNA through mtDNA damage repair (Aamann et al. 2010), nucleoid structural organization (Kamenisch et al. 2010), and mitochondrial transcription (Berquist et al. 2012). Moreover, ERCC6 is involved in mitochondrial dynamics, namely mitophagy (Scheibye‐Knudsen et al. 2012). Accordingly, ERCC6‐deficient cells exhibit deficient mitochondrial BER activity and accumulation of unrepaired oxidative mtDNA lesions (Aamann et al. 2010). Additionally, mtDNA replication machinery is altered in CS‐B cells due to serine protease overexpression, further exacerbating mtDNA instability (Chatre et al. 2015). The absence of ERCC6 also leads to inefficient mitochondrial transcription due to compromised elongation, regardless of the presence of transcription‐blocking lesions in mtDNA (Berquist et al. 2012). The organization of respiratory complexes is altered in ERCC6‐deprived cells, leading to mitochondrial bioenergetic dysfunction, further evidenced by increased oxygen consumption and ROS production (Osenbroch et al. 2009; Scheibye‐Knudsen et al. 2012). Despite these defects, mitochondrial autophagy is defective when ERCC6 is dysfunctional, leading to the accumulation of dysfunctional mitochondria (Scheibye‐Knudsen et al. 2012). Mitochondrial dysfunction will ultimately result in the accumulation of mitochondria with compromised mtDNA integrity and impaired mitochondrial metabolism which is extremely deleterious to the cell.

While the main molecular features of CS‐B have been outlined, a clear comprehensive understanding of how a deficient mechanism influences the progression of other pathways remains elusive. Alterations in mitochondrial function are proposed to arise from nucleus‐mitochondria signaling subsequent to nuclear DNA damage caused by ERCC6 deficiency and PARP1 activation. The accumulation of DNA damage results in the overactivation of PARP1, leading to excessive metabolization of its substrate, NAD^+^ (Boetefuer, Lake, Dreval, et al. 2018; Scheibye‐Knudsen et al. 2014; Thorslund et al. 2005). NAD^+^ regulates several mitochondrial proteins and essential mitochondrial metabolic pathways; thus, NAD^+^ depletion due to increased PARP1 activation will hinder mitochondrial functioning (Okur, Fang, et al. 2020; Scheibye‐Knudsen et al. 2014). Delivery of PARP1 inhibitors and NAD^+^ precursors to a CS mouse model ameliorated the disease phenotype, supporting this hypothesis (Scheibye‐Knudsen et al. 2014). This concept suggests a potential source of mitochondrial dysfunction; however, given that ERCC6 has multiple direct roles in mitochondria, it is unlikely that impairments in the PARP1‐NAD+ axis are the primary cause of mitochondrial deficiencies, but rather a contributory factor.

A potential pathological mechanism underlying the loss of proteostasis in CS has also been suggested, connecting transcriptional impairment with mitochondrial dysfunction. The loss of functional ERCC6 results in impaired RNAP I transcription, causing a disruption in ribosomal metabolism, either through delayed synthesis of new ribosomes or defects in ribosome assembly (Alupei et al. 2018; Qiang et al. 2021). The disruption of ribosomal metabolism reduces protein translation fidelity, resulting in misfolded protein that are prone to oxidation. This, along with the oxidative stress observed in CS‐B cells, leads to increased protein carboxylation. A combination of misfolded proteins, oxidative stress, and the consequent carboxylated proteins, provokes endoplasmic reticulum (ER) stress and unfolded protein response (UPR). The activation of UPR will repress global transcription, further hindering RNAPI transcription. This mechanism leads to loss of proteostasis and instates a feedback‐loop that hinders proteostasis recovery.

## 4 Concluding Remarks

Cockayne Syndrome complementation group B is a complex disorder with diverse underlying molecular mechanisms that contribute for its highly debilitating and multisystemic phenotype. Years of research focusing on the physiological role of ERCC6 have provided extensive knowledge regarding different processes and cellular pathways dependent on ERCC6. Most accumulated knowledge regarding ERCC6 function is related to its crucial role in TC‐NER. Nevertheless, significant advances have been made associating ERCC6 with several other mechanisms that are essential for proper cell functioning. This has helped bridge the gap in the knowledge in the pathological context of Cockayne Syndrome. Currently, ERCC6 has been identified to play an important role in distinct DNA repair mechanism, responsible for tackling different type of DNA damage. These mechanisms include TC‐NER, BER, and DSB repair where ERCC6 is essential for the recruitment of repair machinery, and ICL repair where ERCC6 modulates effector repair factors. Notably, ERCC6 function is not limited to DNA repair. In fact, ERCC6 is implicated in transcription by remodeling chromatin of relevant regions, modulating RNAP I and II and cooperating with transcription factors and co‐factors. Additionally, mitochondrial processes such as mtDNA maintenance, mitochondrial transcription and structural organization also rely on ERCC6. The key role ERCC6 plays in all these cellular processes, highlights the importance of ERCC6 for proper cell functioning. Ultimately, ERCC6 dysfunction leads to extremely deleterious consequences to the cell, which culminates in cellular malfunction and cell death.

Premature aging is a hallmark of progeroid syndromes, such as Cockayne Syndrome. Therefore, a relation between normal aging and Cockayne syndrome pathophysiology may be established to explore the potential mechanisms driving CS progression. Considering the cellular processes ERCC6 is involved in a physiological context, in this review we have organized CS‐B pathophysiology into main three molecular features. These features include DNA damage accumulation, transcriptional dysregulation and mitochondrial dysfunction. Importantly, we consider that these features do not act as isolated pathways but rather influence one another, through a mechanism interplay. This interplay has the potential to exacerbate dysfunction of affected features or induce dysfunction of an otherwise functional feature. Furthermore, in a therapeutic standpoint, exploring CS‐B pathogenesis and potential synergies between pathological mechanism is essential to determine effective therapeutic targets. In line with this, here we propose some interactions that may improve the understanding of the complexity underlying CS‐B pathophysiology. In the future, the interplay between CS‐B‐affected mechanisms should be assessed. This may be done by inducing the impairment of individual mechanisms and evaluating the function of other potentially related cellular processes.

Given that CS‐B presumably arises from a combination of interconnected mechanisms, a therapeutic approach targeting a singular pathological mechanism will be constrained within the broader context of the disorder. Additionally, CS‐B is an autosomal recessive disorder resulting from monogenic mutations in *ERCC6*. These characteristics of CS‐B render *ERCC6* supplementation a straightforward gene therapy approach that would address the underlying cause of this complex disorder and theoretically mitigate all associated disease mechanisms.

The lack of reliable biomarkers for CS‐B, make it challenging to assess CS‐B at a molecular level. Recent efforts have revealed several potential CS biomarkers that will facilitate more precise tracking of disease progression and evaluate the impact of potential therapies at a molecular level. Hyperactivation of NDN has been associated with neuropathological features of CS‐B, especially for neurodevelopmental defects (Liang et al. 2023). In contrast, the downregulation of ATF‐3 responsive gene following genotoxic stress serves as also been proposed to serve biomarker for CS specific phenotype (Epanchintsev et al. 2017). Interestingly, the CS‐specific epigenetic signature may be used to assess the accelerated aging phenotype of CS‐cells (Crochemore et al. 2019).

Relevant advances have been made in the field, however some crucial question that will prove foundational for the mechanisms behind CS‐B to remain to be unveiled. (i) Which pathological mechanism is the main contributor for CS‐B? (ii) Which mechanism is the first to be affected in CS‐B? (iii) Which is the underlying cause for the heterogeneous CS phenotype between patients? Future research will prove essential to tackle these uncertainties and bridge the gap in knowledge regarding CS‐B pathophysiology.

## Author Contributions

Ricardo Afonso‐Reis: original draft preparation. Cristiana R. Madeira: figure and table preparation; review and editing David V.C. Brito and Clévio Nobrega. All authors agreed and approved this article version to be submitted in this journal.

## Conflicts of Interest

The authors declare no conflicts of interest.

## Supporting information


**Table S1.** List of homozygous mutations in *ERCC6*.

## Data Availability

Data sharing is not applicable to this article as no new data were created or analyzed in this study.
