# Supplementary material for: Insights Into Cockayne Syndrome Type B: What Underlies Its Pathogenesis?
Source: Aging Cell. 2025 Jun 19;24(7):e70136. doi: 10.1111/acel.70136 (PMC12266758; doi:10.1111/acel.70136)
Supplement: Supplementary file 1 — Table S1. List of homozygous mutations in ERCC6. [file ACEL-24-e70136-s001.docx]

| Table 1 – List of homozygous mutations in *ERCC6*  *ERCC6*- Homozygous | | | | | |
| --- | --- | --- | --- | --- | --- |
| Mutation | **protein region** | **Patient Code** | **CS type** | **Reference** | **Mutation type** |
| p.Arg683* | ATPase domain | CS789VI | COFS | Laugel et al., 2008 | Premature STOP codon |
| p.Lys1239Glufs*2 | NLS2-CIM | Unknown | COFS | Meira et al., 2000 | Premature STOP codon |
| p.Arg1288* | NLS2-CIM | XVI-1  XVI-2  XIII-6  XIII-7  XIV-2  XIV-6 | COFS | Jaakkola et al., 2010 | Premature STOP codon |
| p.Phe665_Gln723del | ATPase domain | CS11MA | CS II | Calmels et al., 2018 | Deletion |
| p.Val763_Gln794del | ATPase domain | CS514VI CS385VI | CS II | Laugel et al., 2009 | Deletion |
| p.(Asp646Asn) | ATPase domain | CS253ST | CS II | Calmels et al., 2018 | Missense |
| p.Arg670Trp | ATPase domain | Unknown | CS II | Chebly et al., 2018 | Missense |
| p.Leu875Pro | ATPase domain | CS20PV | CS II | Calmels et al., 2018 | Missense |
| p.Trp936Cys | ATPase domain | Unknown | CS II | Wilson et al., 2016 | Missense |
| p.(Thr377Glnfs*28) | AR | CS201ST | CS II | Calmels et al., 2018 | Premature STOP codon |
| p.Lys345Asnfs*24 | Start-AR | CSB1NH | CS II | Falik-Zaccai et al., 2008 | Premature STOP codon |
| p.Arg453* | AR-NLS1 | CS1PV CS3PV | CS II | Colella et al., 1999 | Premature STOP codon |
| p.Lys478Thrfs*9 | NLS1 | CS19PV | CS II | Calmels et al., 2018 | Premature STOP codon |
| p.Trp517* | NLS1-ATPase domain | CS4TAN CS8TAN | CS II | Calmels et al., 2018 | Premature STOP codon |
| p.(Glu564*) | ATPase domain | CS107ST | CS II | Calmels et al., 2018 | Premature STOP codon |
| p.Arg652* | ATPase domain | CS13MA | CS II | Calmels et al., 2018 | Premature STOP codon |
| p.Arg652* | ATPase domain | 08TR4 | CS II | Laugel et al., 2009 | Premature STOP codon |
| p.(Thr659Cysfs*24) | ATPase domain | CS232ST | CS II | Calmels et al., 2018 | Premature STOP codon |
| p.Gly715* | ATPase domain | 08STR1 | CS II | Laugel et al., 2009 | Premature STOP codon |
| p.Asn760Lysfs*2 | ATPase domain | CS8PV | CS II | Calmels et al., 2018 | Premature STOP codon |
| p.(Gln854*) | ATPase domain | CS144ST | CS II | Calmels et al., 2018 | Premature STOP codon |
| p.Gln854* | ATPase domain | CS9MA | CS II | Laugel et al., 2009 | Premature STOP codon |
| p.Met867Thrfs*14 | ATPase domain | CS31PV | CS II | Calmels et al., 2018 | Premature STOP codon |
| p.Ala944Thrfs*10 | ATPase domain | CS11LO | CS II | Calmels et al., 2018 | Premature STOP codon |
| p.Lys971Tryfs*14 | ATPase domain | Unknown | CS II | Kou et al., 2018 | Premature STOP codon |
| p.Gln976Trpfs*70 | ATPase domain | CS23BR | CS II | Calmels et al., 2018 | Premature STOP codon |
| p.(Thr1018Asnfs*32) | ATPase-NLS2 | CS210ST | CS II | Calmels et al., 2018 | Premature STOP codon |
| p.Arg1087* | NLS2-CIM | CS6BI CS8BI | CS II | Laugel et al., 2009 | Premature STOP codon |
| p.Arg1288* | NLS2-CIM | CS817VI | CS II | Laugel et al., 2009 | Premature STOP codon |
| p.[Gln156*; Ser142Asnfs*4] | Start-AR | CS14LO | CS II | Calmels et al., 2018 | Premature STOP codon |
| p.[Arg176*;Gly399Asp; Ser1321Cys] | Start-AR; AR-ATPase domain; NLS2-CIM | Unknown | CS II | Luo et al., 2014 | Premature STOP codon; |
| p.Trp851Arg | ATPase domain | CS3TAN | CS I | Mallery et al., 1998 | Missense |
| p.Pro934Thr | ATPase domain | Unknown | CS I | Wilson et al., 2016 | Missense |
| p.Val957Gly | ATPase domain | CS1IAF | CS I | Mallery et al., 1998 | Missense |
| p.Leu72Cysfs*12 | Start-AR | CS18BR | CS I | Calmels et al., 2018 | Premature STOP codon |
| p.(Glu214*) | Start-AR | CS010ST | CS I | Calmels et al., 2018 | Premature STOP codon |
| p.Glu218Glyfs*4 | Start-AR | AENT4 | CS I | Laugel et al., 2009 | Premature STOP codon |
| p.Ser357* | Start-AR | CS13PV | CS I | Calmels et al., 2018 | Premature STOP codon |
| p.(Ser429Lysfs*7) | AR-NLS1 | CS10LO | CS I | Calmels et al., 2018 | Premature STOP codon |
| p.Gln463* | AR-NLS1 | Unknown | CS I | Zhang et al., 2011 | Premature STOP codon |
| p.Gly715* | ATPase domain | CS23PV | CS I | Calmels et al., 2018 | Premature STOP codon |
| p.Arg735* | ATPase domain | CS17LO CS128ST | CS I | Calmels et al., 2018 | Premature STOP codon |
| p.Arg735* | ATPase domain | CS1TAN | CS I | Mallery et al., 1998 | Premature STOP codon |
| p.(Asp1355Valfs*32) | NLS2-CIM | CS071ST CS222ST | CS I | Calmels et al., 2018 | Premature STOP codon |
| p.Asp1355Valfs*32 | NLS2-CIM | CS1SACT | CS I | Laugel et al., 2009 | Premature STOP codon |
| p.[Arg176*;Ser142Asnfs*4] | Start-AR | CS27PV | CS I | Calmels et al., 2018 | Premature STOP codon |
| p.Arg77Ilefs*6 | Start-AR | KPSX6 | CS III | Hashimoto et al., 2008 | Premature STOP codon |
| p.Glu182Asnfs*4 | Start-AR | CS799VI | CS III | Laugel et al., 2009 | Premature STOP codon |
| p.Trp517* | NLS1-ATPase domain | CS2TAN | CS III | Mallery et al., 1998 | Premature STOP codon |
| p.Arg637Serfs*34 | ATPase domain | Unknown | CS III | Swartz et al., 2015 | Premature STOP codon |
| p.Phe665Tyrfs*18 | ATPase domain | CS1PL | CS III | Calmels et al., 2018 | Premature STOP codon |
| p.(Asp1355Valfs*32) | NLS2-CIM | CS204ST  CS221ST | CS III | Calmels et al., 2018 | Premature STOP codon |
| p.Arg77* | Start-AR | UVS1KO | UVSS | Horibata et al., 2004 | Premature STOP codon |
| p.Arg735* | ATPase domain | GM10905  GM10903 | DSC | Colella et al., 2000 | Premature STOP codon |
| p.Val161Serfs*5 | Start-AR | CS1BR | Unknown | Calmels et al., 2018 | Premature STOP codon |
| p.Arg652* | ATPase domain | CS10X | Unknown | Calmels et al., 2018 | Premature STOP codon |
| p.(Tyr1179Leufs*22) | NLS2-CIM | CS241ST | Unknown | Calmels et al., 2018 | Premature STOP codon |
| p.[Gln156*; Ser142Asnfs*4] | Start-AR | CS3SH | Unknown | Calmels et al., 2018 | Premature STOP codon |

Acidic region (AR); nuclear localization signal (NLS); ERCC8 interaction motif (CIM)

| Table 1 – List of heterozygous mutations in *ERCC6*  *ERCC6*- Heterozygous | | | | | |
| --- | --- | --- | --- | --- | --- |
|  | | | | | |
| Mutation | **protein region** | **Patient Code** | **CS type** | **Reference** | **Mutation type** |
| p.Leu987Pro p.Met752_Gln762del | ATPase domain | CS797VI | COFS | Laugel et al., 2009 | Missense  Deletion |
| p.Leu871Pro p.Lys1172* | ATPase domain; NLS2-CIM | CS881VI | COFS | Laugel et al., 2009 | Missense  Premature STOP codon |
| p.Arg857*  p.Arg1087* | ATPase domain; NLS2-CIM | CS816VI | COFS | Laugel et al., 2008 | Premature STOP codon |
| p.Lys1203fs p.Met867Thrfs*14 | NLS2-CIM; ATPase domain | CS1ABR | CS II | Mallery et al., 1998 | Premature STOP codon |
| p.Tyr510_Arg562del p.Glu608_Gln723del | AR-ATPase; ATPase domain | CS18PV | CS II | Calmels et al., 2018 | Deletion |
| p.0  p.Phe665_Gln723del | ATPase domain | CS10MA; CS1L | CS II | Calmels et al., 2018 | Deletion |
| p.Val724_Gln762del p.Trp686Cys | ATPase domain | CS683VI | CS II | Laugel et al., 2009 | Deletion Missense |
| p.Phe665_Gln723del p.Arg1221* | ATPase domain; NLS2-CIM | CS8MA | CS II | Calmels et al., 2018 | Deletion Premature STOP codon |
| p.Phe665_Gln723del p.Arg1087* | ATPase domain; NLS2-CIM | CS19LO | CS II | Calmels et al., 2018 | Deletion  Premature STOP codon |
| p.Val724_Gln762del p.Ala944Thrfs*10 | ATPase domain | CS1GL | CS II | Laugel et al., 2009 | Deletion Premature STOP codon |
| p.Arg670Trp p.Val763_Gln794del | ATPase domain | CS18LO | CS II | Calmels et al., 2018 | Missense Deletion |
| p.Ser687Leu  p.Arg1288* | ATPase domain; NLS2-CIM | CS278ST | CS II | Calmels et al., 2016 | Missense Premature STOP codon |
| p.Ser687Leu  p.Arg735* | ATPase domain | CS1GGO | CS II | Calmels et al., 2018 | Missense Premature STOP codon |
| p.Arg453*  p.Val763Ilefs*7 | AR-NLS1; ATPase domain | CS1WR | CS II | Calmels et al., 2018 | Premature STOP codon |
| p.0  p.Arg612* | ATPase domain | CS195ST | CS II | Calmels et al., 2018 | Premature STOP codon |
| p.Gly715*  p.Arg857* | ATPase domain | 08STR1 | CS II | Laugel et al., 2009 | Premature STOP codon |
| p.Met867Thrfs*14 p.Lys1198Argfs*4 | ATPase domain; NLS2-CIM | CS117VI | CS II | Laugel et al., 2009 | Premature STOP codon |
| p.Val417Serfs*7  p.0 | AR-NLS1 | CS179VI | CS II | Laugel et al., 2009 | Premature STOP codon |
| p.Gln723*  p.Leu860del | ATPase domain | CS3LE | CS II | Laugel et al., 2009 | Premature STOP codon; Deletion |
| p.Arg683* p.Phe665_Gln723del | ATPase domain | CS12MA | CS II | Calmels et al., 2018 | Premature STOP codon Deletion |
| p.Arg612*  p.Arg975Trp | ATPase domain | Unknown | CS II | He et al., 2017 | Premature STOP codon Missense |
| p.Gln956Argfs*7 p.Ser687Leu | ATPase domain | CS360VI | CS II | Laugel et al., 2009 | Premature STOP codon Missense |
| p.Arg467_Arg562del p.Arg1318Glyfs*12 | NLS1_ATPase domain; NLS2-CIM | CS058ST | CS I | Calmels et al., 2018 | Deletion Premature STOP codon |
| p.Arg670Trp p.Pro1042Leu | ATPase domain; NLS2 | CS2BI | CS I | Mallery et al., 1998 | Missense |
| p.Asp532Gly p.Leu536Trp | ATPase domain | Unknown | CS I | Yu et al., 2014 | Missense |
| p.Trp851Arg p.Arg1318Glyfs*12 | ATPase domain; NLS2-CIM | CS21PV | CS I | Calmels et al., 2018 | Missense Premature STOP codon |
| p.[Gln156;Ser142Asnfs*4] p.Leu471Glnfs*16 | Start-AR; AR-NLS1 | CS1SH | CS I | Calmels et al., 2018 | Premature STOP codon |
| p.[Arg176*;Ser142Asnfs*4] p.Arg652* | Start-AR; ATPase domain | CS1SO | CS I | Calmels et al., 2018 | Premature STOP codon |
| p.Leu700Valfs*60 p.Arg735* | ATPase domain | CS22PV CS28PV | CS I | Calmels et al., 2018 | Premature STOP codon |
| p.Arg735*  p.0 | ATPase domain | CS14PV | CS I | Calmels et al., 2018 | Premature STOP codon |
| p.Gln156* p.His1263Glnfs*67 | Start-AR; NLS2-CIM | CS21BR | CS I | Laugel et al., 2009 | Premature STOP codon |
| p.Trp236* p.Pro500Glnfs*43 | Start-AR; NLS1-ATPase domain | CS493VI | CS I | Laugel et al., 2009 | Premature STOP codon |
| p.Arg735*  p.Arg453* | ATPase domain; AR-NLS1 | 25627 | CS I | Mallery et al., 1998 | Premature STOP codon |
| p.Lys337* p.Tyr834Cysfs*25 | Start-AR; ATPase domain | CS1AN | CS I | Troelstra et al., 1992 | Premature STOP codon |
| p.Arg735*  p.Lys1203fs | ATPase domain; NLS2-CIM | CS2GR | CS I | Calmels et al., 2018 | Premature STOP codon; |
| p.[Arg176*;Ser142Asnfs*4] p.Trp589del | Start-AR; ATPase domain | CS1GO | CS I | Calmels et al., 2018 | Premature STOP codon Deletion |
| p.Arg68Profs*13 p.Asn680Asp | Start-AR; ATPase domain | CS784VI | CS I | Laugel et al., 2009 | Premature STOP codon Missense |
| p.Gln184*  p.Arg670Trp | Start-AR; ATPase domain | CS4BR | CS I | Mallery et al., 1998 | Premature STOP codon Missense |
| p.Phe563Argfs*3  p.[Gln943*; Gln943Profs*8; Asp904_Gln943del] | ATPase domain | CS17PV | CS I/CS II | Calmels et al., 2018 | Premature STOP codon Deletion |
| p.Ser1240_Val1260delinsIle  p.Arg735* | NLS2-CIM; ATPase domain | CS823VI | CS III | Laugel et al., 2009 | Deletion  Premature STOP codon |
| p.Ser142Asnfs*4  p.Arg735* | Start-AR; ATPase domain | CS26PV | CS III | Calmels et al., 2018 | Premature STOP codon |
| p.Arg637Serfs*34 p.Asp749Glufs*4 | ATPase domain | CS543VI | CS III | Laugel et al., 2009 | Premature STOP codon |
| p.Glu182Asnfs*4  p.Glu379* | Start-AR; AR | CS393VI | CS III | Laugel et al., 2009 | Premature STOP codon |
| p.[Gln156*;Ser142Asnfs*4] p.Arg735* | Start-AR; ATPase domain | CS2LE | III? | Calmels et al., 2018 | Premature STOP codon |
| p.0  p.Phe665_Gln723del | ATPase domain | CS2BL | Unknown | Calmels et al., 2018 | Deletion |
| p.Phe665_Gln723del p.Gln953Lys | ATPase domain | CS3BL | Unknown | Calmels et al., 2018 | Deletion Missense |
| p.[Gln156*;Ser142Asnfs*4] p.Arg947* | Start-AR; ATPase domain | CS5MA | Unknown | Calmels et al., 2018 | Premature STOP codon |
| p.Phe563Argfs*3  p.Arg735* | ATPase domain | CS25PV | Unknown | Calmels et al., 2018 | Premature STOP codon |
| p.Arg683*  p.Arg1288* | ATPase domain NLS2-CIM | CS32LO | Unknown | Calmels et al., 2018 | Premature STOP codon |
| p.Ala944Thrfs*10 p.Tyr1179Leufs*22 | ATPase domain  NLS2-CIM | CS1BEL | Unknown | Calmels et al., 2018 | Premature STOP codon |
| p.Ser142Asnfs*4 | Start-AR | Unknown | Unknown | Shehata et al., 2014 | Premature STOP codon |
| p.(Glu215*)  p.(Ser1259*) | Start-AR; NLS2-CIM | Unknown | Unknown | Wu et al., 2016 | Premature STOP codon |
| p.Tyr1179Ilefs*22 p.Phe665_Gln723del | NLS2-CIM; ATPase domain | CS2BE | Unknown | Mallery et al., 1998 | Premature STOP codon Deletion |

Acidic region (AR); nuclear localization signal (NLS); ERCC8 interaction motif (CIM);
